# Multi-Criteria Decision-Making Approach for Pre-Synthesis Selection of the Optimal Physicochemical Properties of TiO_2_ Photocatalytic Nanoparticles for Biomedical and Environmental Applications

**DOI:** 10.3390/molecules29163726

**Published:** 2024-08-06

**Authors:** Nefeli Lagopati, Georgios P. Trachanas, Haris Doukas

**Affiliations:** 1 Laboratory of Biology, Department of Basic Medical Sciences, Medical School, National and Kapodistrian University of Athens, 11527 Athens, Greece; 2 Biomedical Research Foundation, Academy of Athens, 11527 Athens, Greece; 3 Decision Support Systems Laboratory, School of Electrical and Computer Engineering, National Technical University of Athens, 9, Iroon Polytechniou Str., 15773 Athens, Greece

**Keywords:** multi-criteria decision-making, nanoparticles, titanium dioxide, synthesis optimization

## Abstract

Nanomaterials are widely used in several biomedical and environmental applications, due to their ideal properties. However, the synthetic and characterization procedure requires significant costs and has a negative environmental impact. Various methods are available in order to control the pre-synthesis design of the produced materials, predicting their behavior and minimizing the series of experiments. Multi-Criteria Decision-Making is proposed in this study in order to determine the best combination of the physicochemical parameters and to define the best alternative among fifteen different samples of nanostructured titanium dioxide. In particular, the Technique for Order of Preference by Similarity to Ideal Solution (TOPSIS) method was applied to achieve a final ranking of the available alternatives by avoiding several of the trials that would follow testing the biological effect and the photocatalytic degradation of organic pollutants. Thus, this approach helps us to stay environmentally and ethically correct, saving time, money, and energy and also providing an optimization of the nanomaterials that are developed.

## 1. Introduction

The use of nanoparticles has seen exponential growth during the last decades, in the areas of health care, environment, electronics and several other scientific fields, due to their unique physicochemical properties that make them desirable [1,2]. However, the synthesis and characterization of nanomaterials that can be used in biomedical and environmental applications require significant costs, in terms of consumables, reagents, human resources, labor time, and energy [3].

Human pressure on climate change is well established in both the technosphere and civil society. Thus, from an environmental point of view, it is crucial to minimize the global footprint during human activities, even if these are research ones [4]. Laboratory scientists also contribute to climate change, through generally negative impacts on a global level that come not only from the use of natural resources such as water, energy, and materials but also from the consequent production of entropy heat, waste, CO_2_, etc. [5]. In parallel, the ethical aspect of the experimental use of any living organism (e.g., humans, animals, bacteria, etc.) is a significant issue. Thus, the application of those materials that gather the ideal characteristics on any biological system, starting from cell cultures, is obligated in order to avoid any unnecessary trial [6].

Experimentally, the only way to ensure the performance of the produced nanomaterials is to test them. This means that every synthesis process is followed by full characterization of the physicochemical properties and morphology through various spectroscopic, microscopic, and analytical techniques [7]. Thus, by applying common practices, and following the main steps of the scientific method, including trials and experiments, failures, and revisions, it is possible to finally develop an ideal material [8]. In this process, unnecessary tests, or in general, tests that could have been avoided are implemented requiring many hours of equipment use, consumables, and human resources, with an increased environmental, ethical, and financial cost, particularly in terms of productivity [9].

Nanomaterials have been proven to be a promising candidate for biomedical and antipollution applications. Titanium dioxide (TiO_2_) is a very-well-characterized nanomaterial that is widely used in both environmental [10] and biomedical applications [1,2,11]. The physicochemical properties and the photocatalytic potential of TiO_2_, in parallel with its biocompatibility, make it the material of choice for drug-delivery systems as an anticancer agent [11], as well as for wastewater treatment [12]. TiO_2_ can be produced through several methods, such as the sol-gel method [13,14], chemical vapor deposition (CVD) [15], and hydrothermal method [16]. Through different approaches, materials with different characteristics are developed, regarding their morphology (e.g., spherical [13], prismatic, polygonal, etc.), size, stability, crystallinity, charge, dimensionality (e.g., 0D nanoparticles, 1D nanowires/nanotubes [17], 2D films [15], etc. Depending on the different use, TiO_2_ with different properties is selected. For instance, TiO_2_ thin films are used for surface coatings for antibacterial applications [18], nanoparticles for drug delivery systems [19] or wastewater treatments, and nanowires for sensoring [20].

Various physicochemical parameters and morphological characteristics, such as size, zeta potential, hydrodynamic radius (R_h_), specific surface area (SSA), energy band gap, crystal phase, loading capacity, and many others, are crucial to the behavior of a nanomaterial, such as TiO_2_, in case of its use in biomedical or environmental applications [12]. Several methods are available to optimize an experimental procedure during the design and synthesis stage [21,22]. Statistical methods such as the Taguchi Method are among the available approaches to saving money and time, considering research activity from an ethical and ecological point of view [23]. Data mining and artificial intelligence also provide a correlation among the crucial parameters of nanomaterials with similar properties, predicting the biological performance or the potential of the materials to photodegrade pollutants, such as rhodamine B, methylene blue etc. [24].

Multi-Criteria Decision-Making (MCDM) is a well-known method used to determine the best alternative by considering more than one criterion in the selection process [25]. It is considered reliable and accurate, taking into account qualitative and quantitative criteria (with different weights, based on the importance of each criterion in a specific application), that need to be fixed in order to find the best solution [26]. The MCDM method is a user-friendly method that does not require specific or advanced software in order to be applied. A roper classification of the tested parameters is the key point of the process based on the literature, as well as the experience of the researcher. So, MCDM is considered a powerful tool in material science used to predict optimum parameters by providing a ranking of the combination of them.

In this study, MCDM and the Technique for Order of Preference by Similarity to Ideal Solution (TOPSIS) method were applied. This method is a well-known and widely applied technique that allows for selecting the best option among a number of alternatives based on multiple criteria. The main idea of this method is to find solutions that are closest to the ideal solution and farthest from the negative-ideal solution. It is a simple method that provides outputs that are easily understood and explained, and this is why it is preferable in engineering, management, and healthcare. It would be quite difficult to employ this technique to a totally new field of science. In this case, the criteria weights of the tested parameters would be difficult to determine, without previous studies or experience [27].

In this study, the MCDM-TOPSIS method was employed to achieve a final ranking of the alternatives [26] among TiO_2_ samples with different physicochemical properties in order to avoid synthesizing all of them and testing their biological effect and their photocatalytic performance in pollutants’ degradation. This approach will save us time and money and help us to stay environmentally and ethically correct, optimizing our pre-synthesis process. The ultimate goal is to use the ideal nanoparticles that gather the optimal combination of physicochemical properties in biomedical applications, focusing on the anticancer potential as well as the antibacterial potential, and also to use them in applications related to the degradation of organic pollutants. Thus, this ranking would help us to make a list of the materials that can be synthesized as a priority and not to synthesize all of them.

## 2. Results

### Data Numerical Results

In this section, we apply the combination of TOPSIS with Analytic Hierarchy Process (AHP) methods in order to solve the emerged Multi-Criteria Decision Making problem described in Section 4.1. By applying the AHP method described in detail in Section 4.2, we obtain the weights included in Table 1. Table 2 presents the weighted normalized decision table. TOPSIS distances are shown in Table 3. 

The positive and the negative ideal solution are then as follows:

$$p^{+}=(0.006,\;0.007,\;0.081,\;0.019,\;0.122)$$

$$p^{-}=(0.207,\;0.115,\;0.025,\;0.057,\;0.007)$$

Then, the distances from the positive ideal solution and the negative ideal solutions, as well as the relative closeness for each alternative are presented in Table 3.

Based on relative closeness values, we obtain the following ranking for the nanoparticle types:

NP15 > NP9 > NP4 > NP1 > NP2 > NP10 > NP8 > NP7 > NP11 > NP12 > NP13 > NP5 > NP6 > NP14 > NP3

## 3. Discussion

All the samples that are included in this study (15 TiO_2_ samples), have been prepared through similar approaches, and characterized by standard methods, such as dynamic light scattering (DLS), X-ray Diffraction Analysis (XRD), Brunauer-Emmett-Teller (BET), micro-Raman spectroscopy, etc. Focusing on the five more critical parameters that are obtained through these methods, namely size, hydrodynamic radius (R_h_), zeta potential (in absolute values), crystal phase, and Specific Surface Area (SSA), these samples were evaluated.

Given the ranking of the nanomaterials, it is obvious that NP15 presents a very good combination of the parameters that have been considered during the methodology that was applied. More specifically, the 15th sample has a very high SSA (220 m^2^/g), which is a very important parameter of the nanoparticles and is related to an enhanced accessible area for various interactions (physical and chemical) with their surroundings and particularly when internalized within cells [28,29]. Thus, it makes sense that the material with the highest SSA is found in the top positions of the ranking.

Size and SSA are the parameters that are more weighted in the whole procedure, due to their high impact on the final performance of the nanoparticles, the smallest size, the highest bioactivity, and photocatalytic potential of the TiO_2_ nanoparticles [30,31]. Also, the R_h_ gives indirectly a sense of the size of the nanoparticles, and in the case of biomedical and environmental applications, it is important for the R_h_ to be in the range of the nanoscale (1–100 nm) or close to it [32]. Regarding the zeta potential, it is associated with the general stability of the sample, so it is important that the absolute value of this parameter is high [33]. As far as the crystal phase is concerned, TiO_2_ can be found as anatase, as rutile, as brookite, or in a combination of them (also as amorphous material) [34]. Several studies have shown that anatase form is the most bioactive one [1]; thus pure anatase was expected to contribute to the high ranking of the 15th sample, that also has a small size (25 nm), relatively small R_h_ (111.3 nm), adequate value of zeta potential (16.7 mV), and its crystal phase is pure anatase.

The second sample of the ranking is the ninth sample that has an extremely small size and R_h,_ 6 and 67 nm, respectively, pure anatase form, almost the same zeta potential (16.7 mV) as the 15th sample, and lowest SSA (125 m^2^/g). When observing the whole ranking, it is obvious that through this process the best combination of the five criteria emerges, and not each of them alone. Thus, the worst choice of these 15 samples is the third of them according to our results, as it has a size of 100 nm, which is considered high, R_h_ of 349 nm, which also gives a sense of the development of agglomerations, quite good zeta potential (19.1 mV) and anatase form, and very small SSA (15 m^2^/g). Thus, the most crucial parameters, namely size and SSA, are not found in the acceptable range.

So, according to our experimental experience handling those materials, this result seems to be reasonable. Materials, such as the 15th one, have been proven effective in inducing apoptotic cell death to high metastatic breast cancer cells [1] and have been tested also for their capability to photodegrade methylene blue, methyl orange, and rhodamine B, which are common pollutants.

Thus, MCDM, and particularly TOPSIS, seem to be very promising approaches. A bigger database would also enhance this result. Typically, when a scientist starts to study the available literature, in order to find a very good synthesis protocol, they have to decide among 1–5 different studies, so under this point of view, this categorization among 15 different alternatives seems to fit any expectations. Further investigation, based on morphological characteristics of the nanoparticles (spherical, prismatic, flakes, etc.), would also be a new field of study in order to optimize the pre-synthesis procedure.

## 4. Materials and Methods

### 4.1. Data

This section includes the performance of each alternative in each criterion, formulated in a payoff table form (Table 4). In particular, we consider 15 types of TiO_2_ nanoparticles (NP1–NP15) that were previously tested, and these samples are evaluated across five criteria: size, hydrodynamic radius (R_h_), zeta potential (in absolute values), crystal phase, and Specific Surface Area (SSA). These criteria were chosen due to their high impact on the performance of the TiO_2_ nanoparticles. Many previous experimental studies of our team members that were already published in the last decade allow us to categorize the importance of those parameters. Also, a thorough study of other available articles focusing on the same nanoparticles allowed us to draw the same conclusions. Several other parameters, such as energy band gap or crystallinity, are also important for the photocatalytic behavior of these nanoparticles [31,35,36], but these criteria were excluded from the decision table, since in the majority of the published articles, also including those that were chosen, these parameters are almost stable. The crystallinity is very high in all these cases (>80%), and the energy gap ranged among 3–3.1 eV; these slight differences did not affect the final ranking, so there were not included. Also, another important point was that we studied many more studies, but not all of them had a full characterization of the critical parameters, and we decided to choose only those that had studied these five criteria.

The synthesis and properties of the nanomaterials are also generally related to external conditions. These conditions (e.g., temperature, light, pH, etc.) are generally controlled by an experienced scientist, leading to the development of materials with a good repeatability if the synthesis protocol is reverently followed [37]. In this study, experimental data from one of our previous studies were used [1] (sample NP15) in order to use these data as a kind of control sample to test the repeatability of this method. Since we already understand the performance of these nanoparticles, we are sure about their biological and photocatalytic effect, so we can decide about the accuracy of the obtained ranking. Also, several other nanoparticles would be chosen, but for the same reason, ensuring the reliability of this method, TiO_2_ was chosen as a model type of nanoparticles widely used and well-studied.

**Table 4:** Decision table.

Nanoparticle Type	Size (nm)	R_h_ (nm)	Zeta Potential (mV)	Crystal Phase	SSA (m^2^/g)	Refs.
NP1	10	669	16.9	anatase	154	[38]
NP2	20	307	32.3	anatase/rutile (80/20)	73	[38]
NP3	100	349	19.1	anatase	15	[38]
NP4	15	211.4	13.2	anatase	146	[39]
NP5	30	969.3	13.8	anatase	61	[39]
NP6	30	1049	12.3	anatase	61	[39]
NP7	21	256	15.2	anatase/rutile (80/20)	55	[39]
NP8	20	165	18.7	anatase/rutile (80/20)	50	[40]
NP9	6	67	16	anatase	125	[41]
NP10	16	150	18	anatase/rutile (80/20)	55	[41]
NP11	26	190	30	anatase/rutile (80/20)	30	[41]
NP12	38	200	35	anatase/rutile (80/20)	22	[41]
NP13	53	220	28	anatase/rutile (80/20)	18	[41]
NP14	104	490	40	rutile	12	[41]
NP15	25	111.3	16.7	anatase	220	[1]

### 4.2. Methodological Framework

Selection of the best TiO_2_ type defines a classical Multi-Criteria Decision-Making (MCDM) problem [42]. For the needs of the present study, the TOPSIS method was adopted, which offers to the decision maker a final ranking of the alternatives under consideration [27].

TOPSIS is a well-known MCDM technique that helps in selecting the best option among a set of alternatives based on multiple criteria. The core idea is to identify solutions that are closest to the ideal solution and farthest from the negative-ideal solution. TOPSIS is relatively simple to understand and implement and was widely used in various fields such as engineering, management, healthcare, and finance, demonstrating its robustness and adaptability to different decision-making scenarios.

Consider a set of alternatives $A=\left\{A_{1},\ldots ,A_{m}\right\}$ which are evaluates across a set of criteria $C=\left\{C_{1},\ldots ,C_{m}\right\}$ with consequences $x_{ij},i=1,\ldots ,m,\;j=1,\ldots ,n$. Consider also a vector of criteria $w=(w_{1},\ldots ,w_{n})$ expressing the relative importance of each criterion, i.e., it satisfies

$$w_{j}\geq 0,\;j=1,\ldots ,n,\;\sum_{j=1}^{n}w_{j}=1$$

Then, the TOPSIS method includes the following steps.

1. Decision table normalization, according to Equation (1):

(1) $$r_{ij}=\frac{x_{ij}}{\sqrt{\sum_{i=1}^{m}x_{ij}^{2}}}$$

2. Decision table weighting, based on Equation (2):

(2) $$v_{ij}=w_{j}r_{ij}$$

3. Determination of the positive and the negative ideal solution:

$$p^{+}=(p_{1}^{+},\ldots ,p_{n}^{+})=\left\{\left(\max v_{ij}|j\in I\right),\;(\min v_{ij}|j\in J)\right\}$$

$$p^{-}=(p_{1}^{-},\ldots ,p_{n}^{-})=\left\{\left(\min v_{ij}|j\in I\right),\;(\max v_{ij}|j\in J)\right\}$$

where I and J define the benefit and cost type criteria, respectively.

4. Calculate the distance from the positive and the negative ideal solution, defined as follows through Equations (3) and (4):

(3) $$S_{i}^{+}=\sqrt{\sum_{j=1}^{n}{\left(v_{ij}-p_{j}^{+}\right)}^{2}}$$

(4) $$S_{i}^{-}=\sqrt{\sum_{j=1}^{n}{\left(v_{ij}-p_{j}^{-}\right)}^{2}}$$

5. Relative closeness calculation, based on Equation (5):

(5) $$C_{i}=\frac{S_{i}^{-}}{S_{i}^{+}+S_{i}^{-}}$$

6. Rank the alternatives based on $C_{i}$ values (the higher the better).

Step 2 of the TOPSIS method requires the determination of the criteria weights. In this study, the AHP method was adopted in order to determine the relative importance of the criteria [43,44,45]. AHP is a decision-making method, developed by Thomas Saaty in the 1970s at the Wharton School of the University of Pennsylvania, that begins by breaking down decisions into a hierarchical structure of a decision-making goal, criteria, and alternatives [46]. The criteria should be weighted, and the alternatives are scored relative to each other based on the decision-maker performing a series of pairwise comparisons [47]. This weighting and scoring process allows for the generation of a total score for each alternative, by which they are ranked. AHP is widely used for decision-making in many fields, including business, government, engineering, healthcare, and education [48].

Under AHP, the experts’ judgments on the criteria importance, formulated as a pairwise comparison matrix, are aggregated to derive the final weights. In particular, AHP includes the following steps:

1. Definition of the criteria that are included in the study and the alternatives of the decision-making problem.
2. Elicitation of experts’ judgment, in the form of pairwise comparison matrices based on Saaty’s scale (Table 5) [45].
3. Aggregation of individual judgments through the aggregation of the individual Judgment (AIJ) method [43,44].

## 5. Conclusions

The ideal characteristics of nanoparticles make them promising candidates in the field of environmental and biomedical applications. The common procedures are not environmentally friendly due to several trials that are needed in order to obtain nanomaterials well characterized with optimal properties, and these processes require costs, energy, and human resources.

TiO_2_ is a widely used nanostructured material with a great variety of applications. Through the thorough study of this material, it is now well known that some physicochemical parameters, such as size, zeta potential, hydrodynamic radius (R_h_), specific surface area (SSA), and crystal phase play an important role in the bioactivity of TiO_2_ as well as in its capability to degrade pollutants, upon irradiation with visible or ultraviolet light. Various methods are applied to optimize the synthesis procedure and to correlate those parameters.

Multi-Criteria Decision-Making is a reliable method that can provide the best alternative by considering a number of criteria in the selection process. During the present study, MCDM and particularly the TOPSIS method were employed to give us a ranking among 15 different TiO_2_ samples. The obtained ranking highlighted that the 15th sample gathered the best combination of those properties, and in a reasonable way, all the other samples were put in descending order of a predicted efficiency. Particularly, this 15th sample has a small size (25 nm), a very high SSA (220 m^2^/g), small R_h_ (111.3 nm), quite good zeta potential (16.7 mV), and pure anatase as its crystal phase. This method considered all the five criteria and ranked these samples based on the best combination of them.

Thus, the ultimate goal of this study was to find through the MCDM method ideal nanoparticles that gather the optimal physicochemical properties, in order to use them in biomedical and environmental applications. The obtained ranking can help us to prioritize the nanoparticles that should be first developed and tested and not to synthesize all of them.

To sum up, MCDM and particularly TOPSIS seem to be very promising alternative approaches to avoid the synthesis steps of all the proposed samples and test them for their photocatalytic performance and biological effect, thus saving us time, money, energy, and helping us to still respect all the living organisms and environment, minimizing the lab hours, the consumption of lab reagents and cells, and optimizing the pre-synthesis process.

## Figures and Tables

**Table 1:** Criteria weights.

Criterion	Weight ($w_{j}$)
Size	0.252
R_h_ (nm)	0.197
Zeta Potential (mV)	0.181
Crystal Phase	0.164
SSA ($m^{2}/g$)	0.201

**Table 2:** Normalized decision table.

Nanoparticle Type	Size (nm)	R_h_ (nm)	Zeta Potential (mV)	Crystal Phase	SSA ($m^{2}/g$)
NP1	0.01	0.074	0.034	0.019	0.085
NP2	0.021	0.034	0.065	0.057	0.041
NP3	0.207	0.038	0.038	0.019	0.008
NP4	0.016	0.023	0.027	0.019	0.081
NP5	0.031	0.107	0.028	0.019	0.034
NP6	0.031	0.115	0.025	0.019	0.034
NP7	0.022	0.028	0.031	0.057	0.031
NP8	0.021	0.018	0.038	0.057	0.028
NP9	0.006	0.007	0.032	0.019	0.069
NP10	0.017	0.017	0.036	0.057	0.031
NP11	0.027	0.021	0.06	0.057	0.017
NP12	0.039	0.022	0.07	0.057	0.012
NP13	0.055	0.022	0.056	0.057	0.01
NP14	0.108	0.054	0.081	0.038	0.007
NP15	0.026	0.012	0.034	0.019	0.122

**Table 3:** TOPSIS distances.

Nanoparticle Type	$S_{i}^{+}$	$S_{i}^{-}$	$C_{i}$
NP1	0.089	0.219	0.711
NP2	0.096	0.21	0.686
NP3	0.236	0.087	0.269
NP4	0.07	0.228	0.765
NP5	0.145	0.182	0.557
NP6	0.152	0.182	0.544
NP7	0.114	0.206	0.644
NP8	0.112	0.211	0.654
NP9	0.072	0.239	0.77
NP10	0.11	0.216	0.663
NP11	0.117	0.207	0.639
NP12	0.122	0.197	0.617
NP13	0.131	0.181	0.58
NP14	0.162	0.131	0.447
NP15	0.051	0.241	0.825

**Table 5:** Saaty’s scale for pairwise comparison within the AHP method [34].

Intensity	Definition	Explanation
1	Equal importance	Two activities contribute equally to the objective.
3	Moderate importance	Experience and judgment slightly favor one activity over another.
5	Strong importance	Experience and judgment strongly favor one activity over another.
7	Very strong importance	An activity is strongly favored, and its dominance is demonstrated in practice.
9	Extreme importance	Evidence favoring one activity over another is of the highest possible order of affirmation.
2, 4, 6, 8	Intermediate values	When compromise is needed.

## Data Availability

Data are contained within the article.

## Abbreviations

MCDM: Multi-Criteria Decision-Making; TiO2: titanium dioxide; TOPSIS: Technique for Order of Preference by Similarity to Ideal Solution; CO2: carbon dioxide; CVD: chemical vapor deposition; 0D: zero-dimensional; 1D: one-dimensional; 2D: two-dimensional; Rh: hydrodynamic radius; SSA: specific surface area; AHP: Analytic Hierarchy Process; NP: nanoparticle type; DLS: dynamic light scattering; XRD: X-ray Diffraction Analysis; BET: Brunauer–Emmett–Teller.
